# Resistance Assessment for Oxathiapiprolin in *Phytophthora capsici* and the Detection of a Point Mutation (G769W) in PcORP1 that Confers Resistance

**DOI:** 10.3389/fmicb.2016.00615

**Published:** 2016-04-29

**Authors:** Jianqiang Miao, Meng Cai, Xue Dong, Li Liu, Dong Lin, Can Zhang, Zhili Pang, Xili Liu

**Affiliations:** Department of Plant Pathology, China Agricultural UniversityBeijing, China

**Keywords:** oxathiapiprolin, resistance assessment, point mutation, *Phytophthora capsici*, G769W, transformation

## Abstract

The potential for oxathiapiprolin resistance in *Phytophthora capsici* was evaluated. The baseline sensitivities of 175 isolates to oxathiapiprolin were initially determinated and found to conform to a unimodal curve with a mean EC_50_ value of 5.61 × 10^-4^ μg/ml. Twelve stable oxathiapiprolin-resistant mutants were generated by fungicide adaptation in two sensitive isolates, LP3 and HNJZ10. The fitness of the LP3-mutants was found to be similar to or better than that of the parental isolate LP3, while the HNJZ10-mutants were found to have lost the capacity to produce zoospores. Taken together these results suggest that the risk of *P. capsici* developing resistance to oxathiapiprolin is moderate. Comparison of the *PcORP1* genes in the LP3-mutants and wild-type parental isolate, which encode the target protein of oxathiapiprolin, revealed that a heterozygous mutation caused the amino acid substitution G769W. Transformation and expression of the mutated *PcORP1*-769W allele in the sensitive wild-type isolate BYA5 confirmed that the mutation in PcORP1 was responsible for the observed oxathiapiprolin resistance. Finally diagnostic tests including As-PCR and CAPs were developed to detect the oxathiapiprolin resistance resulting from the G769W point mutation in field populations of *P. capsici*.

## Introduction

*Phytophthora capsici* is a highly dynamic and destructive plant oomycete pathogen that causes root, fruit, and foliar diseases in a variety of important vegetable crops including pepper, tomato, eggplant, snap, and lima beans, and all cucurbits (Granke et al., 2012; Lamour et al., 2012), which can lead to devastating crop losses under conducive environmental conditions (Hausbeck and Lamour, 2004; Lamour et al., 2012). In the absence of suitable resistant cultivars, producers rely heavily on synthetic fungicides, which play an important role in the integrated management strategy for *P. capsici* (Lu et al., 2011; Granke et al., 2012). However, the range of synthetic fungicides available for the control of oomycete diseases is limited as these pathogens lack many of the fungicide target sites found in the “true” fungi (Lee et al., 2008; Lu et al., 2010). Furthermore, resistance to the fungicides available has already arisen in many oomycete species including *P. capsici* (Lamour and Hausbeck, 2000; Ishii et al., 2001; Parra and Ristaino, 2001; Café-Filho and Ristaino, 2008; Kousik and Keinath, 2008; Randall et al., 2014). The development of new oomycetes fungicides with high activity and low environmental toxicity is therefore urgently required.

Oxathiapiprolin, 1-[4-[4-[5-(2,6-difluorophenyl)-4,5-dihydro-3-isoxazolyl]-2-thiazoly-l]-1-piperid-inyl]-2-[5-methyl-3-(trifluoromethyl)-1H-pyrazol-1-yl]ethanone, was the first of the piperidinyl thiazole isoxazoline fungicides to be discovered and developed by DuPont (Pasteris et al., 2008, 2016). Oxathiapiprolin has been shown to have substantial activity against a range of plant pathogenic oomycetes, including *Pseudoperonospora cubensis*, *Phytophthora nicotianae*, and *P. capsici* (Ji et al., 2014; Cohen, 2015; Ji and Csinos, 2015), and the results of binding assays and affinity chromatography have shown that the molecular target of oxathiapiprolin is the oxysterol binding protein (OSBP; Andreassi et al., 2013; Pasteris et al., 2016), which is a member of the OSBP-related proteins (ORPs) family. Since the target protein of oxathiapiprolin in *P. capsici* genome has only been annotated with the protein Id: 564296 (PHYCAscaffold_14:545241–548188), and because the function of this protein in *P. capsici* or any other oomycetes remains unknown, the current study refers to this protein as PcORP1.

Since 2012, China has required an assessment of resistance risk for all novel pesticides as part of the regulatory process for the approval of new pesticide products (NY/T 1859.2-2012). The fungicide resistance risk assessment is mainly focused on several factors including the establishment of baseline sensitivities in susceptible pathogens, as well as the selection of resistant mutants, which are subsequently evaluated with regard to their fitness and potential for cross-resistance. Such data is essential in the planning and implementation of anti-resistance strategies intended to prevent or slow the development of fungicide resistance (Grimmer et al., 2014).

In general, mechanisms of fungicide resistance fall into one of five categories: (i) mutation of the target protein, which reduces the binding of the fungicide; (ii) the synthesis of an alternative protein that can substitute for the target protein; (iii) the overexpression of the target protein; (iv) an active eﬄux or reduced uptake of the fungicide; and (v) metabolic breakdown of the fungicide (Ma and Michailides, 2005; Gisi and Sierotzki, 2008; Avenot and Michailides, 2010; Leroux et al., 2010). However, although oxathiapiprolin is in the process of being registered for the control of plant oomycete diseases in China, to date there is no data available regarding the potential of *P. capsici* or any other oomycete pathogen to develop resistance to oxathiapiprolin, or any reports of possible resistance mechanisms. The objectives of the current study were therefore to (i) determine the baseline sensitivity of *P. capsici* to oxathiapiprolin; (ii) assess the risk of resistance to oxathiapiprolin; (iii) investigate oxathiapiprolin-resistance mechanisms in *P. capsici*; and (iv) develop rapid and reliable methods for the detection of oxathiapiprolin-resistant *P. capsici* isolates.

## Materials and Methods

### Fungicides

Both the technical grade oxathiapiprolin (96.7%, active ingredient [a.i.]) and 100 g/l oxathiapiprolin dispersible oil suspension (OD) were kindly provided by Dupont Crop Protection (Wilmington, DE, USA), while the other fungicides used were sourced commercially including dimethomorph (95% a.i., Frey Agrochemicals Ltd., Jiangsu, China), azoxystrobin (98.5% a.i., Syngenta Biotechnology Co., Ltd., Shanghai, China), chlorothalonil (98% a.i., HeNan ChunGuang Agrochemical Co., Ltd., HeNan, China), cyazofamid (96% a.i., Sigma–Aldrich Shanghai Trading Co., Ltd., Shanghai, China), cymoxanil (98% a.i., Xinyi Agrochemicals Company, Jiangsu, China), fluazinam (98.4% a.i., Sigma–Aldrich Shanghai Trading Co., Ltd., Shanghai, China), metalaxyl (96% a.i., Agrolex P. Ltd., Beijing, China), fluopicolide (97% a.i., Bayer CropScience Co., Ltd, Shanghai, China) and zoxamide (97.5% a.i., Gowan Company, USA). Each fungicide was accurately weighed and dissolved in dimethyl sulfoxide (DMSO) to prepare stock solutions (10^4^ μg/ml), which were stored in darkness at 4°C until required.

### *P. capsici* Isolates and Plant Cultivars

The *P. capsici* isolates, 175 in total, were obtained from infected pepper samples collected from 28 provinces throughout China between 2010 and 2013.

Pepper seeds (cv. Xichengdaniujiao) were sown in plastic trays (540 mm × 280 mm, 50 seeds per tray) containing a peat/vermiculite mixture (2:1 v/v). The seedlings were grown in a greenhouse (27 ± 2°C, 80% relative humidity, and 12 h photoperiod) until the pepper plants reached the four-leaf stage.

### Baseline Sensitivity of *P. capsici* to Oxathiapiprolin

The sensitivity of the 175 *P. capsici* isolates to oxathiapiprolin was determined *in vitro* using the mycelia growth assay described in a previous study (Pang et al., 2013). The fungicide concentrations have been listed in **Table 1**, and the final concentration of DMSO in the medium was standardized at 0.1% (v/v). Each treatment consisted of three replicate plates. The diameter of each colony was measured perpendicularly after 5 days incubation at 25°C, and the median effective concentration (EC_50_) for each isolate was calculated according to the formula of a previous study (Pang et al., 2013). Significant differences between the EC_50_ values of individual isolates as well as different populations (isolates from different provinces) were evaluated.

**Table 1:** Concentrations used to determine the sensitivity of *Phytophthora capsici* wild-type isolates and oxathiapiprolin-resistant mutants of to various fungicides.

Fungicide	Concentration (μg/ml)	
	For oxathiapiprolin-sensitive isolates	For oxathiapiprolin-resistant isolates
Oxathiapiprolin	0, 0.0002, 0.0004, 0.0006, 0.0008, 0.001	0, 0.05, 0.2, 0.4, 1, 2
Dimethomorph	0, 0.1, 0.2, 0.4, 0.6, 0.8	0, 0.1, 0.2, 0.4, 0.6, 0.8
Zoxamide	0, 0.02, 0.04, 0.1, 0.4, 1	0, 0.02, 0.04, 0.1, 0.4, 1
Fluopicolide	0, 0.05, 0.1, 0.5, 1, 5	0, 0.05, 0.1, 0.5, 1, 5
Chlorothalonil	0, 0.25, 0.5, 1, 2, 4	0, 0.25, 0.5, 1, 2, 4
Cyazofamid	0, 0.5, 1, 5, 10, 25	0, 0.5, 1, 5, 10, 25
Azoxystrobin	0, 0.1, 0.5, 1, 5, 10	0, 0.1, 0.5, 1, 5, 10
Fluazinam	0, 0.5, 1, 5, 10, 25	0, 0.5, 1, 5, 10, 25
Metalaxyl	0, 0.5, 1, 5, 10, 20	0, 0.5, 1, 5, 10, 20
Cymoxanil	0, 40, 80, 100, 150, 200	0, 40, 80, 100, 150, 200

### Selection of Oxathiapiprolin-Resistant Mutants of *P. capsici*

Twelve *P. capsici* isolates (HD11, HD3, LP3, XS3-1, JN-A-1, HX18, HNJZ10, DY1, DY4, HN-B-8, YY7, LZZH5) were assessed for the adaptation on oxathiapiprolin-amended media. Mycelia plugs (5 mm in diameter) were excised from 5-day-old PDA colonies and transferred to fresh PDA plates containing 0.005 μg/ml oxathiapiprolin, which was tentatively considered the discriminatory concentration for the identification of resistant-isolates. After dark incubation at 25°C for 15–20 days, plates exhibiting hyphal growth were covered with fresh PDA medium containing 0.005 μg/ml oxathiapiprolin. After a further 15–20 days dark incubation at 25°C the surviving isolates were subcultured on fungicide-free PDA and incubated for 5 days before mycelia plugs were transferred to PDA plates containing 0.05, 0.1, or 0.5 μg/ml oxathiapiprolin. After dark incubation at 25°C for 15–20 days, plates exhibiting hyphal growth were again covered with fresh PDA medium containing 0.05, 0.1, or 0.5 μg/ml oxathiapiprolin. The selection procedure was repeated until the resistant colonies exhibited normal rates of growth on PDA amended with 0.05, 0.1, or 0.5 μg/ml oxathiapiprolin.

Putative mutants capable of producing zoospores were purified to single-zoospore isolates using the method of a previous study (Bi et al., 2011).

### Characterization of Oxathiapiprolin-Resistant Mutants

#### (1) Level and Stability of Resistant Mutants

The growth rate of *P. capsici* isolates found to be resistant to oxathiapiprolin were compared to the corresponding parental isolates by culturing them on PDA amended with 0, 0.05, 0.2, 0.4, 1, or 2 μg/ml oxathiapiprolin in darkness at 25°C for 5 days. The level of resistance was described using the resistance factor, which was calculated by dividing the EC_50_ of the resistant strains by the EC_50_ of its parental wild-type isolate.

The stability of the resistance in the mutants was assessed by subculturing them on fungicide-free PDA using mycelial plugs or zoospores. After 10 successive transfers to fresh media, the RFs were calculated for the first and tenth subculture. The “factor of sensitivity change” (FSC) was then calculated by dividing the RF of the tenth subculture by that of the first.

#### (2) Effect of Temperature on Mycelial Growth

The oxathiapiprolin-resistant mutants and corresponding parental isolates were dark-incubated on PDA at 10, 18, 25, 30, and 37°C and the colony diameters were measured perpendicularly after 5 days in the dark. The average of the two measurements was used to compare the mycelial growth of each resistant isolate and its parent. Each treatment consisted of three replicated plates and the experiment was conducted twice.

#### (3) *In Vitro* Sporangium Production and Cystospore Germination

Sporangia and zoospores were obtained according to the protocol of a previous study (Pang et al., 2013). Sporangium production was assayed by counting the number of sporangia per square centimeter of carrot agar. A total of 20 mycelial plugs (5 mm in diameter) were assessed for each isolate: 10 from the culture edge and 10 from the vicinity of the initial inoculum plug, and the entire experiment conducted twice.

Cystospore germination was assessed by plating 100 μl zoospore suspension (10^4^ zoospores/ml) on 1.5% water agar medium. After 12 h dark-incubation at 25°C, the percentage germination of each isolate was estimated by counting 100 cystospores under a light microscope. Cystospores were considered to have germinated if the length of their germ tube was greater than the diameter of the cystospore. The entire experiment was conducted twice.

#### (4) Virulence and Sporangium Production on Detached Pepper Leaves

The virulence and sporangium production of each isolate was assessed using detached pepper leaves (cv. Xichengdaniujiao) according to the protocol of a previous study (Pang et al., 2013).

#### (5) Virulence on Pepper Seedlings

The inoculation of pepper seedlings and disease scoring were performed according to the method of Glosier et al. (2008), with minor modifications. A zoospore suspension was used as the inoculum for LP3 and the LP3-mutants, while a mycelial suspension was used for HNJZ10 and the HNJZ10-mutants. The seedlings were inoculated by adding 2 ml of the zoospore (1 × 10^4^ zoospores/ml) or mycelia (0.02 g/ml) suspension to the soil surface around each seedling. Inoculated plants were incubated in the same greenhouse as described above, and their disease severity rated after 8 days. Each treatment consisted of 30 seedlings.

#### (6) *In Vivo* Control Efficiency of Oxathiapiprolin in Resistant Mutants

A 13.33 μg/ml solution was prepared by diluting oxathiapiprolin (100 g/l, OD) in sterile distilled water and sprayed at a volume of 750 l/ha onto the surface of pepper plants at the four-true-leaf stage. After 24 h, the pepper plants were inoculated with 3 ml zoospore suspension (10^4^ zoospores/ml) of the parental *P. capsici* isolate (LP3) or one of the three resistant isolates (LP3-I, LP3-P, and LP3-M), which was applied to the soil surface near the roots of each plant. The disease severity was rated after 8 days. Each treatment consisted of 25 pepper seedlings. The experiment was performed twice.

#### (7) Cross-resistance

The sensitivity of the 12 oxathiapiprolin-resistant mutants and 21 wild-type *P. capsici* isolates to fungicides with different modes of action (**Table 1**) was determined by measuring their colony diameters after dark-incubation at 25°C for 5 days, and calculating the EC_50_ values as described above. Each combination of isolate and concentration was represented by three replicate plates, and the experiments were performed twice.

### Nucleic Acid Isolation from *P. capsici*

Mycelia were harvested from *P. capsici* isolates grown on PDA for 4 days, and frozen at -80°C until required. Total DNA was isolated using the method of Ristaino et al. (1998). The RNA used to investigate the expression levels of the *PcORP1* gene in oxathiapiprolin-sensitive and -resistant strains was prepared by inoculating 60 ml PDB media with 10 mycelial plugs (5 mm in diameter). The flasks were then incubated at 25°C for 24 h in a rotary shaker at 120 rpm before oxathiapiprolin (0, 0.001, or 1 μg/ml) was added to the treatment flasks. The mycelia were harvested by vacuum filtration after a further 24 h incubation, and frozen at -80°C until required. Total RNA was extracted from the frozen samples using the SV Total RNA Isolation kit (Promega, Beijing, China) and cDNA synthesized using the PrimeScrip RT reagent Kit with gDNA Eraser (Takara Biotechnology Co., Ltd., Dalian, China) or PrimeScript^TM^ II 1st Strand cDNA Synthesis Kit (Takara Biotechnology Co., Ltd., Dalian, China) according to the protocols of the manufacturers.

### Sequencing the *PcORP1* Gene of *P. capsici*

The *PcORP1* sequence documented in the PCT Patent Publication WO13/009971 was used to identify flanking sequences from the *P. capsici* genome v11.0^1^, which were then used to design the primers PcORP1f and PcORP1r (**Table 2**) for the amplification of the full-length *PcORP1* sequence. The PCR was performed in a 50 μl reaction mix containing 50 ng of template DNA, 1 μl of each primer (10 mM), 4 μl of dNTP mixture (2.5 mM each dNTP), 5 μl 10x EasyTaq DNA Polymerase Buffer, and 2.5 U of EasyTaq DNA Polymerase (TransGen Biotech, Beijing, China). The PCR was processed using a MyCycler^TM^ Thermal Cycler (Bio-Rad) with the following program: 94°C for 4 min; followed by 35 cycles of denaturation at 94°C for 30 s, annealing at 58°C for 30 s, and extension at 72°C for 3 min; with a final extension at 72°C for 10 min. The PCR products were sequenced by Beijing Sunbiotech Co. Ltd. and DNAMAN software used to predict and compare the amino acid sequence of the PcORP1 proteins from the wild-type isolates and the oxathiapiprolin-resistant mutants.

**Table 2:** PCR primers used in the study.

Primer	Sequence (5′–3′)	Application
PcORP1f	GCTCCACTTCGCCTCTTT	Amplification of the complete *PcORP1* gene
PcORP1r	TTGCTCTTACCGCTGCTC	
PcORP1-EcoRI	GGAATTCATGCAGGCGCTTCAGGACGC	Amplification of the complete ORF of *PcORP1*
PcORP1-SacII	TCCCCGCGGCTAATGCCCAGCCGAACTGC	
pTOR-F1	CCAAGTCCCAACCGACTCTT	Primers for validating the transformants
pTOR-R1	GTTCTACAAACGGCCTTCTT	
RT- PcORP1f	CACCACAGTATCGGACAGC	Real-time quantification of *PcORP1* expression
RT-PcORP1r	CAAACCCAGCAATGGAGTA	
Pc-actinf	ACTGCACGTTCCAGACGATC	
Pc-actinr	CCACCACCTTGATCTTCATG	
AS-PcORP1f3	TATGCTCAACACCAACAATT	Allele-specific PCR
AS-PcORP1r3	CCTTCCACTCTTGCGATT	
Pc-CAPsf	CACCTACATTACCGACCTGG	Cleaved amplified polymorphic sequence
Pc-CAPsr	CCACTCTTGCGATTCGTC	

### Transformation of *P. capsici*

The complete coding sequence of *PcORP1* was amplified from the cDNA of the wild-type LP3 isolate and the oxathiapiprolin-resistant mutant LP3-M using the primers PcORP1-EcoRI-F and PcORP1-SacII-R (**Table 2**) and ligated into the pTOR (Whisson et al., 2007) expression vector as *Eco*RI/*Sac* II fragments. The sequences of the insertions were verified by DNA sequencing before transforming the oxathiapiprolin-sensitive wild-type isolate BYA5 according to the protocol of a previous study (Dou et al., 2008). The transformants produced were screened by PCR using genomic DNA and cDNA as a template and the primers pTOR-F1 and pTOR-R1 (**Table 2**) to confirm the presence and expression of the transgenes, respectively.

### SYBR Green Real-time RT-PCR Assay

The real-time PCR was performed using the ABI7500 sequence detection system (Applied Biosystems) and the SYBR Premix DimerEraser kit (Takara Biotechnology Co., Ltd., Dalian, China) with the primers listed in **Table 2** and the protocol of the manufacturer. The relative quantities of the PCR products were calculated using the 2^-ΔΔCt^ method and the *actin* gene (Yan and Liou, 2006) as a reference to normalize the quantification of *PcORP1* expression. The entire experiment was conducted three times.

### Molecular Detection of the Oxathiapiprolin-Resistance Mutation *PcORP1*-769W by Allele-Specific PCR (AS-PCR) and Cleaved Amplified Polymorphic Sequences (CAPs)

An allele-specific primer set, AS-PcORP1f3/AS-PcORP1r3 (**Table 2**) was designed to the single nucleotide base change from G to T in the codon at position 769 of the *PcORP1* gene, and its specificity was determined using oxathiapiprolin-resistant mutants (LP3-H, LP3-F, LP3-K, LP3-M, LP3-I, LP3-M) and sensitive wild-type isolates (HD11, LP3, YY7, HNJZ10). The PCR was performed as described above but with an annealing temperature of 54.4°C and a shorter extension period of 1 min. After the PCR was completed, a 5-μl aliquot of the PCR product from each sample was analyzed by electrophoresis using a 1.5% agarose gel.

The single point mutation at codon 769 in the *PcORP1* gene of the LP3-mutants was also found to create a recognition site for the restriction endonuclease *Pf1*MI that was suitable for CAPS analysis. A primer set, PcCAPsf/PcCAPsr (**Table 2**) was designed to amplify a 1261 bp fragment of the *PcORP1* gene containing the point mutation. PCR analysis using the total DNA from six oxathiapiprolin-resistant and four oxathiapiprolin-sensitive isolates was performed as described above using total DNA as a template, an annealing temperature of 60°C and an extension period of 1.5 min. The amplified fragments were then digested with *Pf1*MI (Thermo Scientific, Beijing, China) according to the protocol of the manufacturer and analyzed by electrophoresis using a 1.5% agarose gel.

### Statistical Analysis

The data generated in the study were analyzed using DPS software ver. 7.05. Differences between the means were determined using Duncan’s multiple range test at *P* = 0.05.

## Results

### Baseline Sensitivity of *P. capsici* to Oxathiapiprolin

**FIGURE 1:**
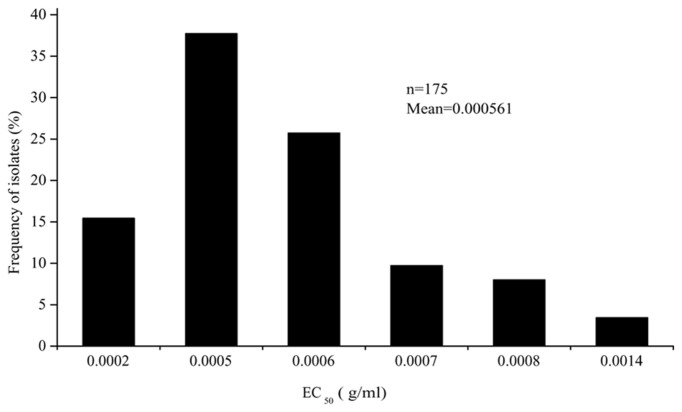
**Frequency distribution of the EC_50_ values for oxathiapiprolin in 175 *Phytophthora capsici* isolates originating from 28 provinces throughout China**.

The EC_50_ values of the 175 *P. capsici* isolates tested ranged from 3.19 × 10^-4^ to 9.86 × 10^-4^ μg/ml oxathiapiprolin, and produced a unimodal distribution with a mean of 5.61 × 10^-4^ μg/ml (**Figure 1**). The narrow range and low EC_50_ values of the *P. capsici* isolates indicated that there was no preexisting oxathiapiprolin-resistance. Furthermore, the data revealed that there was no correlation between the sensitivity of the isolates and their geographic location as a range of EC_50_ values was observed in all of the different populations (**Table 3**).

**Table 3:** Sensitivity to oxathiapiprolin of 175 *Phytophthora capsici* isolates originating from 28 provinces throughout China.

Location^a^	Number of isolates	EC_50_ (×10^-4^μg/ml)	
		Range	Mean^b^
Heilongjiang	11	4.57–8.92	6.28 b–f
Jilin	6	5.02–8.38	5.86 c–g
Liaoning	4	5.32–7.12	6.19 b–f
Tianjin	8	3.76–5.43	4.86 f–h
Neimenggu	3	6.80–7.79	7.54 a–c
Beijing	7	3.97–5.59	4.82 f–h
Hebei	7	4.66–6.62	5.89 c–g
Henan	8	3.97–8.38	5.09 e–h
Shandong	7	4.41–9.86	5.95 b–g
Gansu	8	3.93–6.45	5.09 e–h
Shanxi	3	4.01–4.94	4.37 gh
Xinjiang	7	5.19–7.74	6.41 a–f
Jiangsu	6	4.45–5.03	4.68 f–h
Anhui	4	4.97–6.64	5.87 c–g
Sichuan	11	3.92–8.03	5.53 d–h
Xizang	6	4.42–7.07	5.71 d–g
Hubei	9	3.64–7.16	5.22 e–h
Hunan	4	6.37–7.78	6.72 a–e
Zhejiang	9	3.59–6.25	4.33 gh
Jiangxi	4	4.67–8.86	7.13 a–d
Guizhou	3	7.28–9.28	7.98 a
Yunnan	12	3.19–8.54	5.39 e–h
Fujian	6	5.73–9.66	7.61 ab
Guangdong	4	3.59–3.99	3.85 h
Guangxi	6	4.32–7.48	5.36 e–h
Hainan	8	3.85–9.51	5.64 d–g
Qinghai	2	4.40–4.45	–
Taiwan	2	4.62–5.90	–

*^a^Province where isolates were collected.*

*^b^Values followed by the same letter within a column do not differ significantly (P < 0.05).*

### Generation of *P. capsici* Mutants Resistant to Oxathiapiprolin

Although 12 parental varieties were assessed, only two, LP3 and HNJZ10, were found to produce mutants when exposed to oxathiapiprolin. In total, 12 oxathiapiprolin-resistant mutants were recovered, all with resistance factors (RF) > 300 (**Table 4**).

**Table 4:** Stability and level of oxathiapiprolin resistance of two groups of *Phytophthora capsici* mutants and their parental isolates, LP3 and HNJZ10, after the first and tenth subculture on fungicide-free medium.

Isolate^a^	Origin	EC_50_(μg/ml)		RF^b^		FSC^c^
		First	Tenth	First	Tenth	
LP3	Parent	3.70 × 10^-4^	6.05 × 10^-4^	–	–	–
LP3-F	Mutant	0.37	0.72	1000.00	1190.08	1.19
LP3-H	Mutant	0.35	0.58	945.95	958.68	1.01
LP3-I	Mutant	0.41	0.53	1108.11	876.03	0.79
LP3-K	Mutant	0.37	0.61	1000.00	1008.26	1.01
LP3-M	Mutant	0.40	0.65	1081.08	1074.38	0.99
LP3-P	Mutant	0.42	0.67	1135.14	1107.44	0.98
HNJZ10	Parent	4.96 × 10^-4^	4.75 × 10^-4^	–	–	–
HNJZ10-A	Mutant	0.53	0.67	1068.55	1410.53	1.32
HNJZ10-C	Mutant	0.52	0.23	1048.39	484.21	0.46
HNJZ10-E	Mutant	0.61	0.73	1229.84	1536.84	1.25
HNJZ10-F	Mutant	0.17	0.19	342.74	400.00	1.17
HNJZ10-G	Mutant	0.16	0.35	322.58	736.84	2.28
HNJZ10-H	Mutant	0.39	0.22	786.29	463.16	0.59

^a^LP3 and HNJZ10 correspond to the wild-type parental isolates. LP3 and its mutants were transferred by zoospores and HNJZ10 and its mutants by mycelia plugs. ^b^RF, resistance factor; ratio of EC_50_ of fungicide-resistant mutants relative to the EC_50_ of the parental isolate. ^c^FSC, factor of sensitivity change; the ratio of RF of the tenth transfer to the RF of the first transfer.

### Characterization of Oxathiapiprolin-Resistant Mutants

#### (1) Resistance Factor and Stability

The initial RFs of the oxathiapiprolin-resistant mutants ranged from 322.58 to 1135.14 (**Table 4**) and although subculturing through ten asexual generations on fungicide free media altered the RFs slightly, all remained above 300. The six mutants derived from LP3 were subcultured by zoospores and showed increased RFs except for LP3-I, while the six mutants obtained from HNJZ10 were subcultured by mycelia plugs, and generally resulted in increased resistance with exceptions of HNJZ10-C and HNJZ10-H.

#### (2) Effect of Temperature on Mycelial Growth

The optimal temperature for mycelial growth was found to be 25°C for all the *P. capsici* isolates tested (**Table 5**). In addition, it was noted that the LP3-mutants grew at the same rate or faster than the parental isolate at 10, 18, 25, 30, and 37°C (with the exceptions of LP3-H, LP3-M, and LP3-P at 37°C), which indicating that the LP3-mutants exhibited enhanced growth at all the temperatures tested. However, the HNJZ10-mutants grew at the same rate or slower than the parental isolate irrespective of temperature, which indicated that the mutation had a negative effect on the growth of this group of mutants.

**Table 5:** Mycelial growth of two groups of oxathiapiprolin-resistant *Phytophthora capsici* mutants and their parental isolates on potato dextrose agar at various temperatures.

Isolate	Colony diameter (mm)^a^				
	10°C	18°C	25°C	30°C	37°C
LP3	5.7 ab	30.3 e	46.0 e	44.2 e	22.2 b
LP3-H	7.2 a	38.8 cd	58.5 c	51.5 c	12.2 d
LP3-F	5.3 b	42.7 a	58.8 bc	53.3 b	27.8 a
LP3-K	6.3 ab	40.3 bc	58.7 bc	53.0 bc	22.7 b
LP3-M	6.0 ab	37.0 d	56.8 d	46.3 d	13.7 d
LP3-I	6.0 ab	41.7 ab	62.7 a	60.0 a	31.5 a
LP3-P	5.2 b	42.5 ab	59.5 b	53.7 b	17.8 c
HNJZ10	7.2 a	27.3 ab	49.8 a	44.8 a	11.7 a
HNJZ10-A	2.0 b	30.0 a	50.0 a	35.3 c	0.0 c
HNJZ10-C	2.0 b	25.0 b	38.5 c	34.0 c	0.3 c
HNJZ10-E	0.2 b	24.5 b	43.2 b	30.5 d	1.5 c
HNJZ10-F	5.5 a	24.8 b	38.0 c	40.7 b	3.8 b
HNJZ10-G	1.5 b	20.0 c	32.7 e	29.8 d	0.0 c
HNJZ10-H	5.5 a	25.2 b	34.8 d	39.5 b	0.0 c

*^a^Colony diameters were measured 5 days post inoculation. Values followed by the same letter within a column do not differ significantly (P < 0.05).*

#### (3) Sporangium Production and Cystospore Germination

The sporangia production and cystospore germination of the LP3-mutants were similar with that of the parental isolate LP3 in either the *in vitro* tests or on detached bell pepper leaves, except for the LP3-K, which produced significantly more sporangia than the wild-type LP3 *in vitro* (**Table 6**). In contrast, the HNJZ10-mutants were found to produce far fewer sporangia than the parental isolate in both the *in vitro* test and on detached bell pepper leaves, and in many cases failed to produce any sporangia at all. Furthermore the HNJZ10-mutants that did produce a small number of sporangia, including HNJZ10-A, HNJZ10-F, and HNJZ10-G, all failed to produce zoospores (**Table 6**).

**Table 6:** Fitness of two groups of oxathiapiprolin-resistant *Phytophthora capsici* mutants compared to their sensitive parental isolates^a^.

Isolate	*In vitro*		*In vivo*^b^		
	No. sporangia(×10^4^/cm^2^)	Cystospore germination (%)	Lesion area (cm^2^)	No. Sporangia (×10^4^/cm^2^)	Disease score
LP3	0.61 b	98.60 a	11.98 a	0.61 ab	0.60 a
LP3-F	0.64 b	98.36 a	10.95 a	0.56 ab	0.55 a
LP3-H	0.79 ab	97.82 a	16.01 a	0.75 ab	0.54 a
LP3-I	0.57 b	98.69 a	11.35 a	0.71 ab	0.55 a
LP3-K	1.08 a	100.00 a	11.33 a	0.52 b	0.55 a
LP3-M	0.40 b	98.87 a	11.76 a	0.83 a	0.53 a
LP3-P	0.42 b	99.54 a	10.91 a	0.65 ab	0.55 a
HNJZ10	0.62 a	97.68	9.11 a	0.52 a	0.47 a
HNJZ10-A	0.06 bc	–^c^	1.31 b	0.00 b	0.06 b
HNJZ10-C	0.00 c	–	1.76 b	0.00 b	0.07 b
HNJZ10-E	0.00 c	–	0.00 b	0.00 b	0.00 b
HNJZ10-F	0.01 c	–	0.00 b	0.00 b	0.00b
HNJZ10-G	0.18 b	–	1.91 b	0.00 b	0.05 b
HNJZ10-H	0.00 c	–	0.00 b	0.00 b	0.00 b

*^a^Values followed by the same letter within a column do not differ significantly (P < 0.05). ^b^Lesion area and sporangia production were determined on detached pepper leaves, while the disease score was determined on living pepper plants. ^c^Zoospores of the HNJZ10-mutants could not be recovered.*

#### (4) Virulence on Detached Bell Pepper Leaves and on Pepper Plants

Both the LP3-mutants and parental isolate were found to be virulent and caused typical and severe symptoms on both detached bell pepper leaves and whole plants. Furthermore, no significant difference was detected between the pathogenicity of the parental isolate LP3 and the LP3-mutants. In contrast, the pathogenicity of HNJZ10-mutants was dramatically reduced compared to the parental isolate, with three of the mutants, HNJZ10-E, HNJZ10-F, and HNJZ10-H, completely lacking virulence in both assays (**Table 6**). Having stronger survival traits, only the LP3-mutants were selected for further investigation.

#### (5) Control of LP3-Mutants and LP3 on Pepper Plants Treated with Oxathiapiprolin

Oxathiapiprolin provided substantially better control of the wild-type isolate LP3 than the resistant mutants LP3-M, LP3-I, and LP3-P at 10 g/ha with control efficiencies of 73.32, 13.95, 29.98, and 23.72%, respectively.

#### (6) Cross-resistance

**FIGURE 2:**
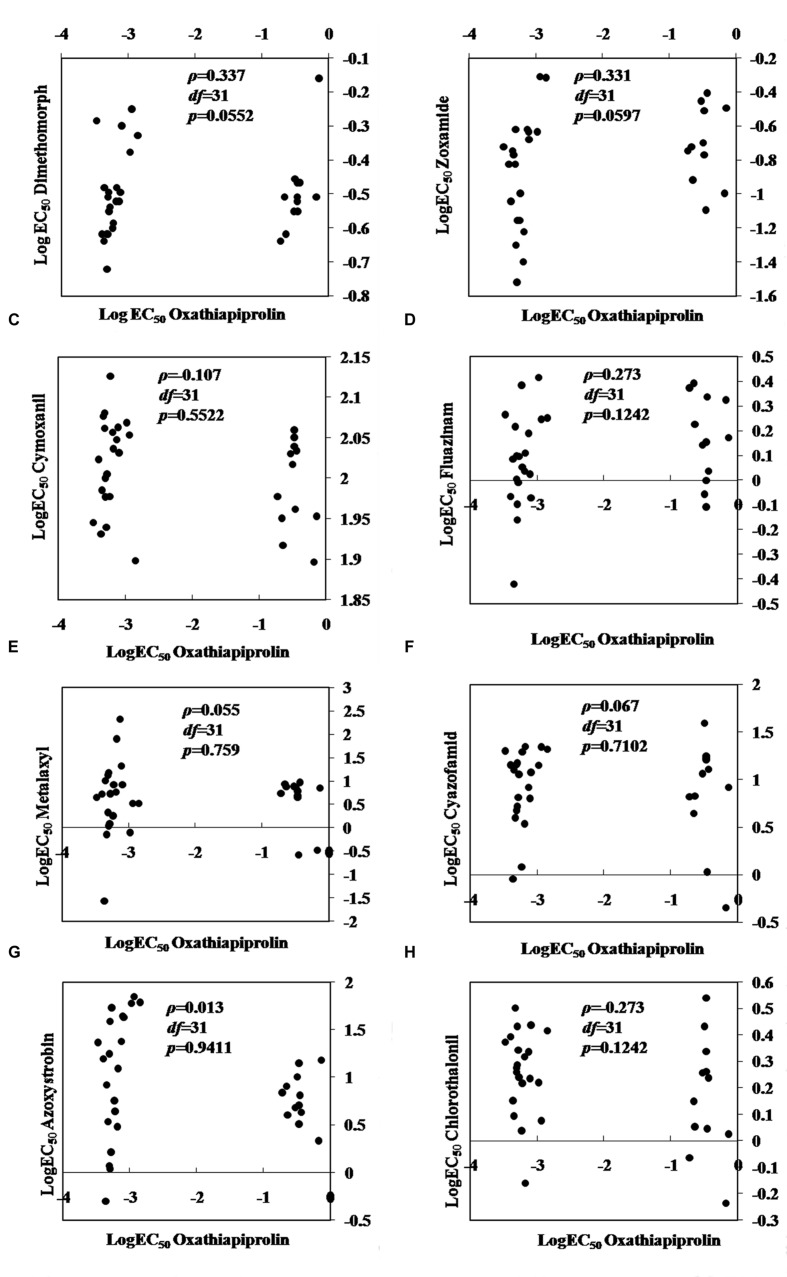
**Spearman’s rank correlation coefficients for cross-resistance between oxathiapiprolin and other oomycete fungicides in *Phytophthora capsici*. (A)** dimethomorph; **(B)** zoxamide; **(C)** cymoxanil; **(D)** fluazinam; **(E)** metalaxyl; **(F)** cyazofamid; **(G)** azoxystrobin; **(H)** chlorothalonil.

No cross-resistance was detected between oxathiapiprolin and any of the other common oomycete fungicides tested including dimethomorph, zoxamide, chlorothalonil, cyazofamid, azoxystrobin, fluazinam, metalaxyl, and cymoxanil (*P* > 0.05; **Figure 2**).

### Cloning and Analysis of the *PcORP1* Gene from the Parental Wild-type Isolates and Oxathiapiprolin-Resistant Mutants of *P. capsici*

**FIGURE 3:**
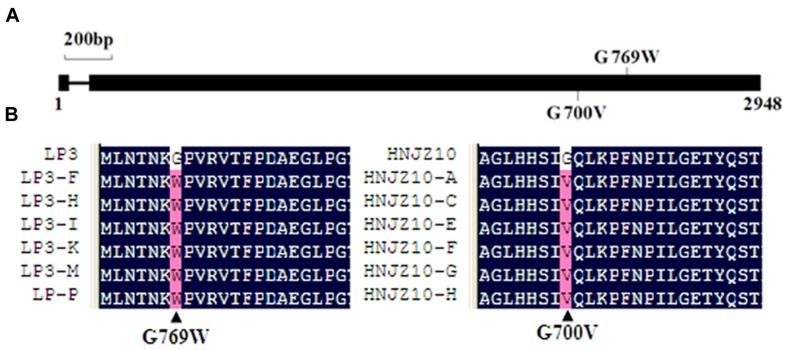
**Multiple sequence alignment indicating the site of the point mutations in the PcORP1 protein that result in oxathiapiprolin resistance. (A)** Structure of the *PcORP1* gene. Numbers represent the size in base pairs. Point mutations in oxathiapiprolin-resistant mutants and the predicted amino acid substitution in the mutant gene products are indicated. **(B)** Alignment of partial amino acid sequences of PcORP1 protein in *Phytophthora capsici* mutants and their sensitive parental isolates, LP3 and HNJZ10.

The full-length nucleotide sequences of the *PcORP1* gene was 2948 bp and contained one 74 bp intron after nucleotide 54 (PHYCAscaffold_14:545241-548188, **Figure 3A**). The *PcORP1* gene was predicted to encode a 957 amino acid polypeptide chain with a molecular weight of 104.13 kDa. Sequence analysis of the wild-type isolates and the resistant mutants revealed two separate heterozygous point mutations. In the case of the six LP3-mutants the mutation occurred in codon 769 and resulted in the amino acid glycine (GGG) being replaced by tryptophan (TGG), while the mutation in the HNJZ10-mutants resulted in a different glycine (GGA) at codon 700 being replaced by valine (GTA; **Figure 3B**).

### Expression Levels of *PcORP1* in the LP3-Mutants and LP3 Parental Isolate in the Presence or Absence of Oxathiapiprolin

**FIGURE 4:**
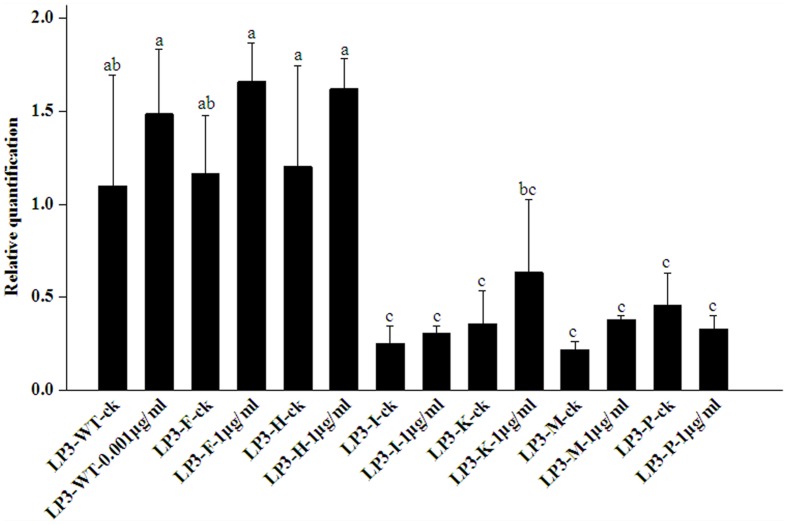
**Expression levels of *PcORP1* in the LP3 wild-type (LP3-WT) and six LP3-mutants (LP3-F, -H, -I, -K, -M, and -P) in the presence or absence (-ck) of oxathiapiprolin.** Expression levels were calibrated to the LP3 wild-type in the absence of the fungicide (LP3-WT-ck, value of 1). Different letters above the columns indicate statistically significant differences (*P* < 0.05).

All the isolates had no significantly altered *PcORP1* expression in the absence of oxathiapiprolin. Compared to the wild-type isolate LP3, the LP3-F and LP3-H mutants had no obviously different expression whether oxathiapiprolin was present or absent (**Figure 4**). In contrast, the other four isolates LP3-I, LP3-K, LP3-M, and LP3-P had significantly reduced expression irrespective of whether the fungicide was present or absent, and their expression levels changed little under the two treatments (**Figure 4**).

### Back-Transformation of *PcORP1*, and the Expression Level of *PcORP1* in the Transformants

**FIGURE 5:**
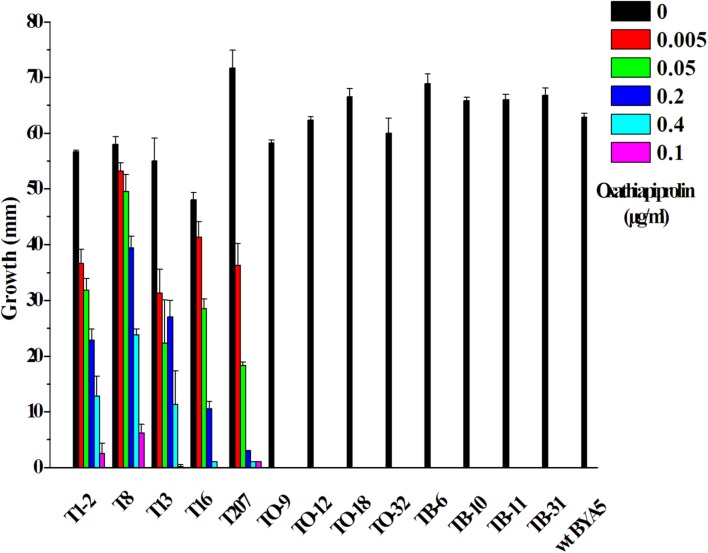
**Oxathiapiprolin sensitivity of *P. capsici* transformants and the wild-type isolate BYA5.** The effect of increasing oxathiapiprolin concentration on the growth of the wild-type isolate BYA5 and two groups of transformant expressing either the wild-type *PcORP1* gene (TO) or the mutated version *PcORP1*-769W (T) as well as a third group transformed with an empty vector (TB).

Vectors containing the wild-type (*PcORP1*-769G) or mutated type (*PcORP1*-769W from LP3-M) allele of *PcORP1* were used to transform the oxathiapiprolin-sensitive wild-type isolate BYA5, respectively. In total, 13 stable transformants were recovered: five containing the G769W mutation (T1-2, T8, T13, T16, and T207), four containing the wild-type *PcORP1*-769G allele (TO-9, TO-12, TO-18, and TO-32) and four control transformants containing the empty vector (TB-6, TB-10, TB-11, and TB-31). Assays assessing their mycelia growth rate on potato dextrose agar (PDA) amended with oxathiapiprolin revealed that all of the transformants containing the mutated *PcORP1* allele (*PcORP1*-769W) exhibited reduced levels of sensitivity to oxathiapiprolin (**Figure 5**), while the transformants containing the wild-type allele (*PcORP1*-769G) or the empty vector remained sensitive and were unable to grow at 0.005 μg/ml oxathiapiprolin (**Figure 5**).

**FIGURE 6:**
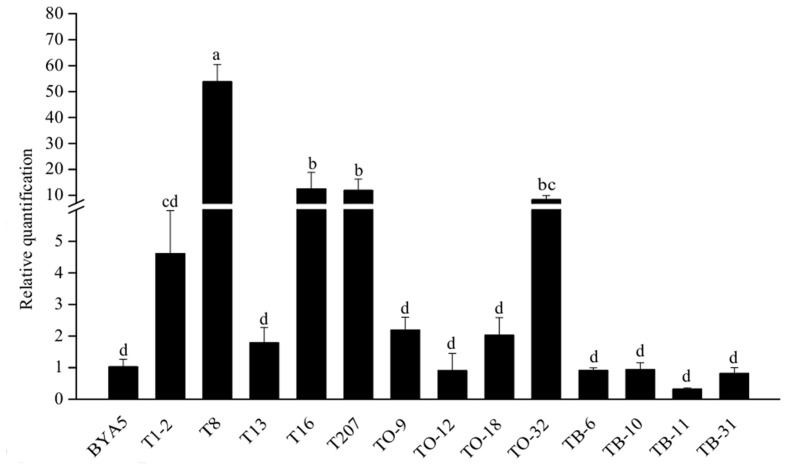
**Expression levels of *PcORP1* in the wild-type BYA5 and two groups of transformant expressing either the wild-type *PcORP1* gene (TO) or the mutated version *PcORP1*-769W (T) as well as a third group transformed with an empty vector (TB).** Expression levels were calibrated to the wild-type (BYA5, value of 1). Different letters above the columns indicate statistically significant differences (*P* < 0.05).

With the exception of TO-12, TB-6, TB-10, TB-11, TB-31, all the other transformants had increased levels of *PcORP1* expression. However, only four of which (T8, T16, T207, TO-32) differed significantly from the untransformed wild-type BYA5 (**Figure 6**).

### Allele-Specific PCR (AS-PCR) and Cleaved Amplified Polymorphic Sequence (CAPs) for the Rapid Detection of Oxathiapiprolin-Resistant *P. capsici* Isolates with a Point Mutation G769W in their *PcORP1* Genes

**FIGURE 7:**
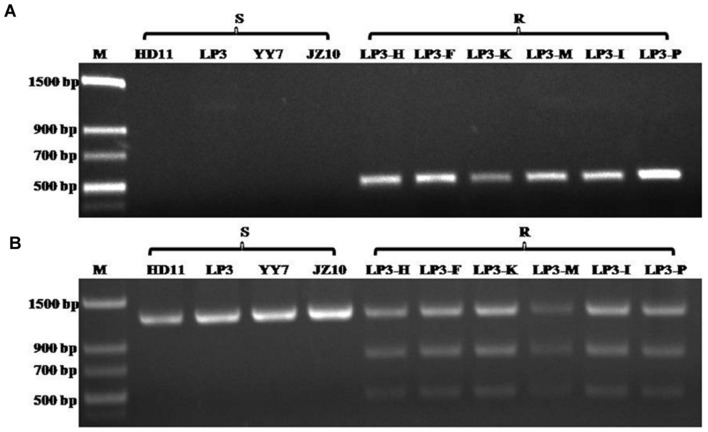
**Results of the Allele-specific PCR (A) and Cleaved amplified polymorphic sequence analyses (B) confirming their specificity for the G769W mutation in the *PcORP1* gene of *Phytophthora capsici*: Lane 1, Molecular marker; Lanes 2–5, Sensitive wild-type isolates; Lanes 6–11, LP3-mutants that exhibit good fitness and high resistance to oxathiapiprolin**.

Although the HNJZ10-mutants exhibited a high degree of resistance to oxathiapiprolin, their fitness was limited by their inability to produce zoospores. The development of detection methods for oxathiapiprolin resistance in *P. capsici* was therefore focused on the LP3-mutants. Two separate molecular methods were developed for the rapid detection of the point mutation at codon 769 in the *PcORP1* gene: AS-PCR and CAPs. It was found that AS-PCR using the primer pair AS-PcORP1f3/AS-PcORP1r3 (**Table 2**) and an annealing temperature of 54.4°C amplified a conspicuous 494-bp fragment from the oxathiapiprolin-resistant mutants (LP3-F, LP3-H, LP3-I, LP3-K, LP3-M, and LP3-P), but not from any of the oxathiapiprolin-sensitive isolates (HD11, YY7, LP3, and JZ10; **Figure 7A**).

The CAPs approach took advantage of the cleavage site for the restriction enzyme *Pfl*MI (5′-CCANNNN↓NTGG-3′) that was created by the point mutation in codon 769 (GGG→TGG), and allowed the discrimination of the wild-type and mutant isolates according to their specific restriction profiles. The technique required a preliminary amplification of a *PcORP1* fragment containing the cleavage site using the primer set Pc-CAPsf/Pc-CAPsr (**Table 2**) followed by digestion with *Pfl*MI. As expected, all of the sensitive isolates produced a single band (1261 bp) while the resistant mutants containing the heterozygous mutation in codon 769 GGG→TGG produced two additional bands of 789 and 472 bp (**Figure 7B**).

## Discussion

The first step in monitoring and managing the development of fungicide resistance in the field is the establishment of a baseline sensitivity for pathogen populations, which requires a large number of isolates to provide an accurate measurement (Russell, 2004). To date, there has only been one other report describing the sensitivity of *P. capsici* to oxathiapiprolin that only focused on a limited number of isolates (Ji and Csinos, 2015), so the current study of 175 field isolates from 28 provinces throughout China has provided valuable data regarding the sensitivity of wild populations of this pathogen. Given that oxathiapiprolin has never been applied to the fields of China, the EC_50_ values obtained provide a very accurate measure of the baseline sensitivity of *P. capsici* to oxathiapiprolin in China. The ratio of the highest to lowest EC_50_ values was only 3.09, indicating a low degree of variation in sensitivity among isolates. Furthermore, the low EC_50_ values (the mean of 5.61 × 10^-4^ μg/ml) and unimodal distribution provide strong evidence that no oxathiapiprolin-resistant subpopulations exist in wild populations of *P. capsici*. The results of the current study can therefore be used to provide a baseline for monitoring changes in the sensitivity of *P. capsici* populations to oxathiapiprolin.

Although oxathiapiprolin-resistant *P. capsici* mutants and *P*. *infestans* mutants had been reported in previous literatures (Andreassi et al., 2013; Pasteris et al., 2016), but the current study provides the first report of oxathiapiprolin-resistant *P. capsici* mutants resulting from fungicide adaptation. The resistant mutants obtained by screening mycelial plugs on fungicide-amended media provided a model for how resistance could develop naturally in the field. However, it should be noted that the development of resistance only occurred in two of the 12 isolates tested. This might be explained by the genotypic variation among different *P. capsici* populations, as it has been documented in China that significant levels of genotypic variation are produced following the sexual and asexual reproduction of this species (Hu et al., 2013a,b).

The characteristics of resistant isolates are also an important factor when evaluating fungicide resistance risks. While the fitness of the HNJZ10-mutants was found to be significantly impaired by their reduced number of sporangia and incapacity to produce zoospores, the LP3-mutants exhibited strong adaptive traits in a range of developmental stages, including mycelial growth, sporangium production, cystospore germination, and most importantly, pathogenicity. This adaptability suggests that the LP3-mutants would remain competitive, successfully infecting plants and having a high rate of survival in nature, and indicates that this kind of oxathiapiprolin-resistant subpopulation could successfully reproduce, become established and dominate the field population in response to the selection pressure of oxathiapiprolin.

Analysis of the *PcORP1* expression levels in the five LP3-mutants revealed that treatment with oxathiapiprolin did not induce any significant changes in the expression of the target protein, while sensitivity assays confirmed that there was no cross-resistance between oxathiapiprolin and other fungicides. Taken together these results provide strong evidence that the resistance mechanism of the LP3-mutants is unlikely to be conferred to the multi-drug resistance (MDR) usually associated with the activity of ABC and MFS transporters (Ziogas et al., 2006; Gulshan and Moye-Rowley, 2007). In plant pathogens, the MDR was well investigated in *Botrytis cinerea*. In 2008, the frequency of *B*. *cinerea* MDR strains from French and German wine-growing regions was 38%. The MDR strains showed considerable resistance levels toward fludioxonil, fenhexamid, fyprodinil, farbendazim, foscalid, etc, and the phenotypes are caused by mutations leading to overexpression of eﬄux transporters (Kretschmer et al., 2009). Nakaune et al. (1998) reported that demethylation inhibitor resistant strains of the plant pathogenic fungus *Penicillium digitatum* were shown to be simultaneously resistant to cycloheximide, 4-nitroquinoline-*N*-oxide and acriflavine. *PMR1* gene encoding an ATP-binding cassette (ABC) transporter was involved in the MDR of *P*. *digitatum* (Nakaune et al., 1998).

Comparison of the *PcORP1* genes in the LP3-mutants and wild-type parental isolate revealed a heterozygous mutation that caused the substitution of tryptophan for the glycine at amino acid 769. The role of the mutated *PcORP1*-769W in oxathiapiprolin resistance was investigated by transformation of the mutated allele into the sensitive isolate BYA5. The five transformants recovered all had reduced sensitivity to oxathiapiprolin, although to a lesser degree than the LP3 mutants that resulted from fungicide adaptation. However, it should be noted that the transformants retained both their wild-type alleles and were therefore likely to still be producing PcORP1 proteins sensitive to oxathiapiprolin, which could have compromised the level of shift in sensitivity resulting from the transgene. This hypothesis is supported by the observation that the total expression level of *PcORP1* was not significant altered in the majority of the transformants (T1-2, T13, T16, T207, TO-9, TO-12, TO-18, and TO-32) compared to the wild-type BYA5. Furthermore the observation that several transformants (TO-9, TO-12, TO-18, and TO-32) expressing the wild-type transgene (*PcORP1*-769G) exhibited no change in their oxathiapiprolin sensitivity. Taken together these results provide strong evidence that the mutated allele *PcORP1*-769W was the causes of the oxathiapiprolin resistance observed in the LP3 mutants.

Previous research has shown that several different point mutations in the *PiORP1* gene (GenBank accession number XP_002902250.1) of *P. infestans*, which were obtained though by ultraviolet mutagenesis, were likely to be conferred to oxathiapiprolin resistance, including L733W; S768I, F, K, or Y; G770A, I, P, V, L; N837I, F or Y; G839W; P861H; L863W or F; and I877F or Y (Andreassi et al., 2013). It was noted that amino acid sequence alignment revealed that PcORP1 has 81.96% similarity to PiORP1, and that the G700V in the HNJZ10-mutants and the G769W mutation in the LP3-mutants were equivalent to the 770 and 839 residues in PiORP1. Although the HNJZ10-mutants with the G700V point mutation exhibited strong levels of resistance, it was also found that they had significantly reduced the fitness. We hypothesize that the fitness penalty (sporangia, zoospore, and lesion area) observed in G700V mutants might contribute to the inability of the pathogen to develop stable oxathiapiprolin-resistant field populations. In contrast, the LP3-mutants with the point mutation G769W exhibited robust levels of fitness. All fitness parameters were found to be similar to or better than that of the parental isolate LP3. The ability of *P. capsici* to generate spontaneous oxathiapiprolin-resistant mutants coupled with raised fitness of G769W mutants can suggest this target site mutation would emerge as an oxathiapiprolin-resistance determinant in *P. capsici* field populations worldwide. Further research is also required to discover whether there are other point mutation in *PcORP1*, including those analogous to the ones occurring in *PiORP1*, also result in oxathiapiprolin resistance in field.

Fungicide resistance can be separated into two categories depending on whether the resistance is controlled by a single gene or by several genes (Gisi and Staehle-Csech, 1988). Pathogen populations exhibiting monogenic resistance are characterized by the occurrence of several distinct resistant subpopulation that usually having a high RF values (>100), as typified by the mandipropamid resistance of *Plasmopara viticola* (Blum et al., 2010). In contrast, polygenic resistance is usually associated with a continuous distribution of fungicide sensitivity and lower RF values, as observed in resistance to demethylation inhibitors (Gisi et al., 2000). The current study revealed two types of separate stable mutations in the LP3-mutants and HNJZ10-mutants, respectively, which resulted in RF values greater than 300. Molecular analysis confirmed that the resistance resulted from separate point mutations in single alleles of the *PcORP1* gene, which encodes the target protein of oxathiapiprolin. The heterozygous nature of the genotype in all the mutants assessed indicated that the resistance might be controlled by single dominant genes, which infers that these resistant *P. capsici* isolates could have a high chance of developing in the field, and that sexual recombination would not affect the spread of the resistant population as dominant or semi-dominant genes are always expressed. Further research including genetic characterization experiments is required to substantiate these preliminary results.

The strong survival characteristics of the LP3-mutants indicate that it is extremely important to monitor the G769W mutation in field populations of *P. capsici*. The AS-PCR and CAPs techniques developed in the current study were both effective in detecting oxathiapiprolin resistance (G769W) in the *P. capsici* mutants. The AS-PCR primers were designed and optimized by mismatching the tyrosine in the position of the second nucleotide at the 3′-end of the forward primer according to the method of a previous report (Chen et al., 2012). Compared to conventional methods of detecting fungicide-resistance, the molecular methods described here are much quicker and allow detection of the oxathiapiprolin-resistant phenotype in as little as 5 h by direct sampling of diseased tissue. Although resistance based on G769W has not yet been detected in the field, the methods developed in this study will serve as an important tool to monitor the development of point mutations in the *PcORP1* gene that confer resistance to oxathiapiprolin in *P. capsici*.

The high activity and single target site of oxathiapiprolin combined with the monogenic resistance and high fitness of some resistant mutants indicates that the inherent risk of oxathiapiprolin is high. Combining the low inherent risk of *P*. *capsici*, we estimate that there is a moderate risk of oxathiapiprolin resistance developing in field populations of *P. capsici* according to the grading standards of Brent and Hollomon (2007). The absence of cross-resistance between oxathiapiprolin and other anti-oomycete fungicides suggests that measures to limit the number of applications per season and the application of oxathiapiprolin in mixtures with other anti-oomycete fungicides could help prevent the rapid development of resistance in field populations.

## Author Contributions

XL, JM, MC, and ZP designed the experiments. JM, MC, XD, and DL performed the *in vitro* and *in vivo* experiments. JM, MC, LL, and CZ performed the molecular biology experiments. JM and MC interpreted the results and XL and JM wrote the paper. All authors reviewed the manuscript.

## Conflict of Interest Statement

The authors declare that the research was conducted in the absence of any commercial or financial relationships that could be construed as a potential conflict of interest.

## Footnotes

1 http://genome.jgi-psf.org/Phyca11/Phyca11.home.html
